# Loss of chromatin modulator Dpy30 compromises proliferation and differentiation of postnatal neural stem cells

**DOI:** 10.1093/jmcb/mjz041

**Published:** 2019-05-24

**Authors:** Ting Zhao, Yan Hong, Guo-li Ming, Hongjun Song

**Affiliations:** Department of Neuroscience and Mahoney Institute for Neurosciences, Perelman School of Medicine, University of Pennsylvania, Philadelphia, PA 19104, USA

Epigenetic regulation via chromatin modulation plays pivotal roles in regulating neural stem cells (NSCs) both during embryonic development and in adult neurogenesis (Yao et al., 2016). One classic epigenetic mechanism is covalent post-translational modifications to histone proteins, including methylation, phosphorylation, acetylation, ubiquitination, and sumoylation. In particular, methylation of histone H3 at K4 and K27 positions act antagonistically to maintain active and repressed gene expression states, respectively. Although it is established that gene expression regulated by H3K27 methylation is one of the major determinants of the capacity of NSCs for either self-renewal or lineage differentiation, little is known about the role of H3K4 methylation in NSC regulation (Albert and Huttner, 2018). In the current issue, Shah et al. (2019) show that Dpy30 and H3K4 methylation are essential for proliferation and differentiation of postnatal NSCs.

H3K4 methylation, catalyzed by methyltransferases, is a conserved feature of active genes. In mammalian cells, there are six H3K4 methyltransferases that share a highly conserved catalytic SET domain (SET1A, SET1B, MLL1, MLL2, MLL3, and MLL4). SET1/MLL enzymes function towards H3K4 methylation in the context of a multi-subunit complex, which contains WDR5, RbBP5, Ash2l, and Dpy30 as integral core subunits (Hyun et al., 2017). A previous study explored whether H3K4 methyltransferases regulate neurogenesis by deleting *MLL1* in NSCs at embryonic day 13.5 (E13.5) and found compromised neuronal differentiation in the postnatal subventricular zone (SVZ) due to dysregulation of *Dlx2* expression, a key regulator of SVZ neurogenesis (Lim et al., 2009). Surprisingly, instead of reducing H3K4 tri-methylation, *MLL1* deletion resulted in increased deposition of H3K27 tri-methylation at the *Dlx2* promoter, which converted the state of *Dlx2* gene from ‘active’ to ‘repressed’. Therefore, defective postnatal neurogenesis caused by *MLL1* deficiency seems to be irrelevant to H3K4 methylation.

**Figure 1:**
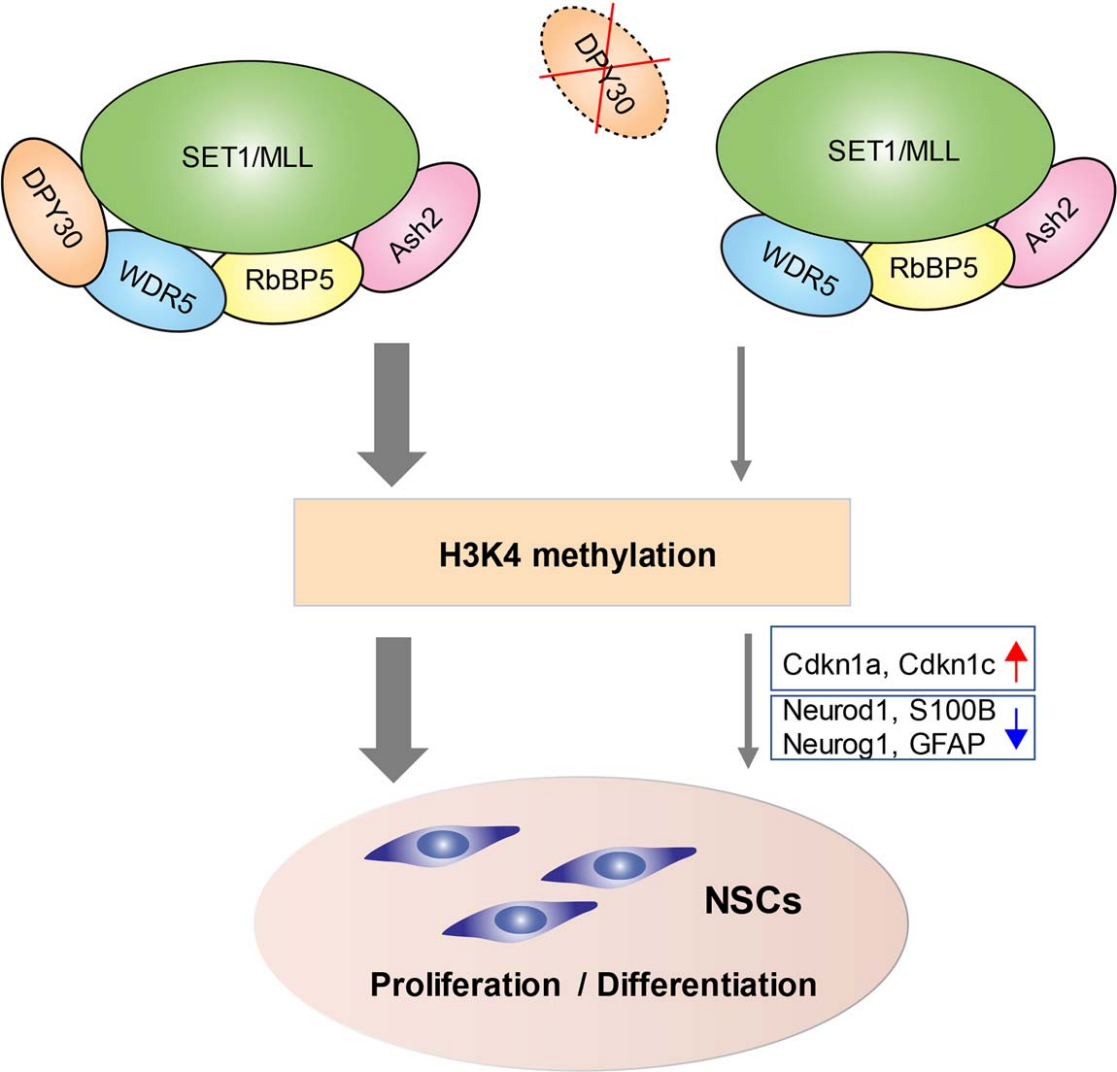
Schematic of impaired proliferation and differentiation of postnatal NSCs caused by the loss of *Dpy30* and reduction of H3K4 methylation.

In the current study, Shah et al. (2019) silenced Dpy30 expression in NSCs from E13.5 using a Cre-loxP system. Dpy30 knockout (KO) pups exhibited growth retardation and ataxia and died during postnatal days 20–27 (P20–27). Moreover, the loss of Dpy30 resulted in enlarged lateral ventricles, deformed dentate gyrus (DG), and a reduction in cerebellar folia, accompanied by global H3K4 methylation reduction (Figure 1). Similar to the SVZ, DG is also a neurogenic region in the mammalian postnatal brain (Ming and Song, 2011). Using DG and SVZ tissues dissected from P12 KO mice, transcriptome analysis revealed downregulation of neuronal markers in the DG and astrocytic markers in the SVZ of KO mice. These results suggest perturbed neurogenesis and gliogenesis selectively in KO DG and SVZ, respectively, although we cannot rule out the probability that potential apoptosis induced by the loss of Dpy30 contributes to these changes.

To further explore the impact of Dpy30 loss on neurogenesis and gliogenesis, Shah et al. (2019) performed immunostaining and found a reduced number of NSCs in the postnatal KO DG and SVZ. The astrocytic population decreased in both KO DG and SVZ, and the neuronal decrease was found specifically in KO DG. Furthermore, Shah et al. (2019) applied an *in vitro* serial replating assay to investigate the proliferative capacity of NSCs collected from SVZ. KO NSCs exhibited a reduced ability to form neurospheres compared to control NSCs, and no viable KO cells were observed after several passages, supporting the model that Dpy30 is required for NSC self-renewal in a cell-autonomous manner. Finally, using short hairpin RNA (shRNA) to knock down Dpy30 in mouse and human neural progenitor cell (NPC) lines and then inducing them to differentiate, Shah et al. (2019) found inefficient neuronal and glial differentiation of Dpy30-knockdown NPCs, suggesting important roles of Dpy30 and H3K4 methylation in the fate determination of NSCs.

What is the molecular mechanism underlying proliferation and differentiation deficits in Dpy30-deficient NSCs? Cyclin-dependent kinases (CDKs) are serine/threonine kinases important for regulating cell cycle and proliferation (Lim and Kaldis, 2013). Upregulation of CDK inhibitors, such as *Cdkn1a* and *Cdkn1c,* were found in the SVZ in a subset of KO mice, which may account for the impaired proliferation of KO NSCs. On the other hand, in Dpy30-knockdown NPCs, fate-determining genes, such as *NEUROD1*, *NEUROG1, GFAP,* and *S100B,* failed to be induced after differentiation, suggesting that the expression of these lineage-specific genes is regulated by Dpy30-dependent H3K4 methylation.

The most striking finding in this study is that *Dpy30* KO mice fail to form the subgranular zone, a neurogenic niche harboring NSCs that give rise to neurons and astrocytes in the adult DG (Ming and Song, 2011). Beginning around E13.5 in rodents, dentate precursors, which originate from dentate neuroepithelium, migrate towards the nascent hippocampal fissure to form the primitive dentate structure (Bayer and Altman, 1974). Dentate NSCs settle into the subgranular zone around P7 for continuous postnatal dentate neurogenesis throughout life (Berg et al. 2019). How Dpy30 loss perturbs embryonic and neonatal DG development and consequently leads to the loss of the postnatal neurogenic niche in the DG remains unknown and can be investigated in the future by conditional *Dpy30* deletion at different developmental stages in the dentate lineage. While the current study provides direct *in vitro* evidence for the role of Dpy30 and H3K4 methylation in regulating NSC self-renewal and differentiation, future studies using the inducible KO animals can address their roles and mechanisms in regulating postnatal and adult neurogenesis in both DG and SVZ *in vivo*.

In summary, Shah et al. (2019) identified Dpy30 and associated H3K4 methylation as important regulators of proliferation and differentiation of postnatal NSCs (Figure 1). Growing evidence suggests an association between deficient H3K4 methylation and mental disorders. For example, loss-of-function mutations in *SETD1A*, a H3K4 methyltransferase, was identified in patients with schizophrenia and developmental brain disorders (Singh et al., 2016). Given the pivotal roles of H3K4 methylation in the brain development, findings from the current study may have implications for understanding the etiology of neurodevelopmental disorders.
